# The Effectiveness of a Resilience Intervention Program on Emotional Intelligence of Adolescent Students with Hearing Loss

**DOI:** 10.3390/children6030048

**Published:** 2019-03-21

**Authors:** Narges Adibsereshki, Nikta Hatamizadeh, Firoozeh Sajedi, Anoshirvan Kazemnejad

**Affiliations:** 1 Department of Psychology and Education of Exceptional Children, University of Social Welfare and Rehabilitation Sciences, Tehran 1985713834, Iran; n.adib@hotmail.com; 2 Pediatric Neurorehabilitation Research Center, University of Social Welfare and Rehabilitation Sciences, Tehran 1985713834, Iran; fisajedi@gmail.com; 3 Department of Biostatistics, Faculty of Medical Sciences, Tarbiat Modares University, Tehran 14115-331, Iran; kazem_an@modares.ac.ir

**Keywords:** emotional intelligence, hearing loss, resilience, intervention

## Abstract

Adolescents with hearing loss have been shown to have some emotional difficulties. This study investigated the effectiveness of a resilience training program on the emotional intelligence of mainstreamed adolescent students with hearing loss. In this experimental study, a pre-test, post-test, follow up and control group design was implemented. After receiving informed consents, 122 students with hearing loss in mainstreaming settings were randomly assigned to intervention and control groups (61 students in interventional group and 61 in control). The interventional group received training in groups of 3 to 5, for 6 weeks (two times per week for 75 min). The intervention focused on feelings, thinking (positive, negative) and outcomes of negative thinking, coping strategies, strengths and weakness, problem solving, communication skills, social skills, negotiation, despising and ridiculing, intra- and interpersonal skills. The Connor-Davidson resilience scale (CD-RISC) and the Schutte Emotional Intelligence Scale were used to measure the resilience and the emotional intelligence (EI) of participants respectively just before intervention, as well as at the 6- and 14-week marks. The Friedman Test was used to compare changes in emotional intelligence between interventional and control groups. The intervention increased the resilience scores by 20 points. Although the EI of both groups were similar at the beginning of the research, there was a significant difference between the control and interventional groups in emotional intelligence and its aspects after intervention, at the 6-week and 14-week measurements (*p* < 0.001). The effect size was 1.5 for the EI total score. The 6-week resilience training program was very effective at improving emotional intelligence and could be readily used to help students with hearing loss improve their emotional abilities.

## 1. Introduction

Emotional Intelligence (EI), consisting of a group of related mental and cognitive abilities, offers solutions to education, work and health problems. It plays a major role in developing an individual’s personal and social capabilities. Mayer and Salovy described emotional intelligence as the ability to perceive, integrate, understand, and regulate emotions to promote personal growth [1]. Emotional, personal, and social abilities are the basis of emotional intelligence [2].

There has been a growing interest in EI among adolescents over recent years. Adolescents may face adjustment difficulties and have potential for alienation [3]. This age group, as students, may face various challenges in achieving academic success, managing relationships with peers and transitioning grades or changing schools (e.g., primary to middle school to high school). While these situations tend to generate stress for all adolescents, students with disabilities experience even more challenges and stress. High emotional intelligence can lead to easing of the problems and success; on the other hand more, worries and concerns in students are correlated with lower emotional intelligence [4]. 

The emotional abilities of individuals with hearing loss are influenced by their unique developmental path, and they usually experience more socio-emotional risks than their hearing peers [5]. Hearing loss affects language and communication abilities, which results in emotional and social developmental deviations [6]. The emotional abilities in children with hearing loss is impacted by a variety of factors, including severity of hearing loss, early hearing assessment and intervention, hearing status of the family members, and mode of communication use by the family with the child with hearing loss [7]. It has been found that children and adolescents with hearing loss have problems with building social relationships and managing their own emotions [8,9]. Therefore, they may experience more stress [10]. On the other hand, resiliency is a key resource for managing the stress of everyday life [11,12] and helps in bouncing back following adversity [12,13]. 

Resilience has been described in various disciplines, mostly in psychology, psychiatry, education, and pediatrics. Based on Developmental Systems Theory, resilience is determined as the capacity of a system to adapt successfully to challenges that intimidate the function, durability, and development of the system [11,12,13]. For an individual, resilience reflects all the adaptive capacity available at a given time in a given context through many different processes and connections. Humans have a great capacity for adaptation to adversity; actually, their resilience depends on many factors, including their relationships with other people who could help them in this process [14].

Resilience and emotional intelligence influence each other and are tools for defeating stressful and difficult situations. People with higher emotional intelligence show less distress in stressful situations [15,16]. Resilient individuals have optimistic and energetic approaches to life and have high positive emotionality. Positive emotions in turn produce valuable outcomes in the coping process. Individuals who have the emotional knowledge necessary to cope can use this in stressful situations. Indeed, people can recognize their own emotions, can build relationships, make complex decisions, develop conflict-management skills and bounce back from adverse experiences [17,18,19,20,21]. Researchers note that individuals with high resiliency can effectively regulate their emotions [22,23]. Some programs have addressed resilience promotion in adolescents, and their effectiveness in improving resilience has been reported [24,25,26]. However, the effects of those interventions on the emotional intelligence of adolescents have not been investigated. Thus, the present study aimed to examine the effects of a resilience promotion program on emotional intelligence of adolescent students with hearing loss.

## 2. Methods

This was an experimental study with a pre-test, post-test, follow up and control group design.

### 2.1. Participants

**Figure 1:**
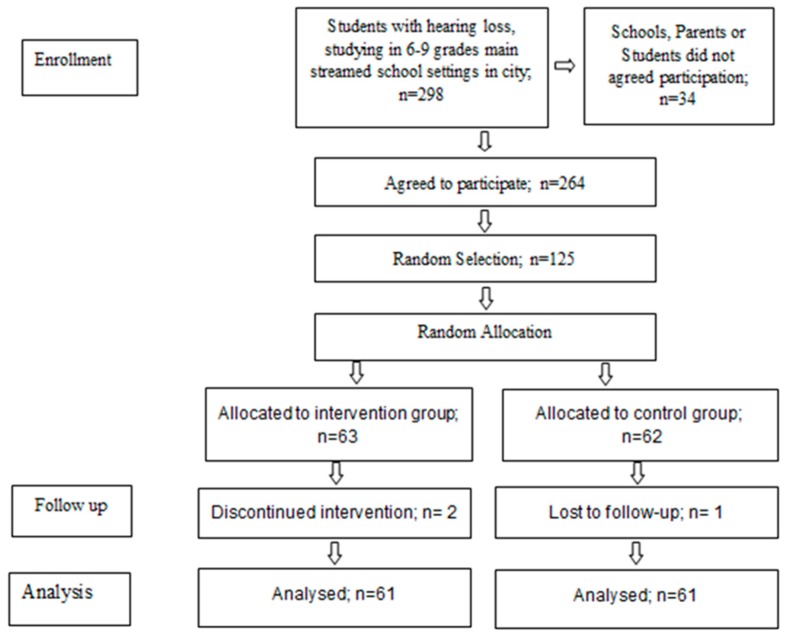
Flow diagram.

298 students were originally introduced by the Education Ministry to be considered for participation in the study, all in 6th to 9th grades. Of the 298 students, 34 were not included because the schools, parents or students themselves did not agree to participation in the study. Of the 264 who agreed to participate, 125 were randomly selected to include in the study. After obtaining informed parental and student consent, the participants were randomly assigned to experimental and control groups. This is presented in Figure 1.

The ethics committee of the University of Social Welfare and Rehabilitation Sciences approved this study (Ethical approval number: IR.USWR.REC. 1396.212).

### 2.2. Measurements

#### 2.2.1. Resilience Scale 

The Connor-Davidson resilience scale (CD-RISC) [27] used in this research consists of 25 items in which each item is rated as not always true = 4, often true = 3, sometimes true = 2, rarely true = 1, and never true = 0. The total score range is between 0 and 100. Higher scores indicate a greater degree of resilience. In this study, the Cronbach’s alpha was calculated to be 0.88.

#### 2.2.2. Emotional Intelligence Scale

The Schutte Emotional Intelligence Scale [28] used in this study consists of three subscales: Emotion Regulation, Emotion Appraisal and Expression, and Emotion Utilization. The scale consists of 33 items, in which each item is rated as 1 = strongly disagree, 2 = disagree, 3 = neither agree nor disagree, 4 = somewhat agree, 5 = strongly agree. After reversing the scores of items 5, 28, 33, the total scores can be calculated by the summation of scores. The total score ranges from 5 to 165. In the present sample, the Cronbach’s alphas were calculated to be 0.83 for total EI, 0.72 for Emotion Regulation, 0.61 for Emotion Appraisal and Expression, and 0.47 for Emotion Utilization. 

A brief questionnaire was used to gather demographic data on age, gender, grade, severity of hearing loss and using hearing assistive devices.

Pre-test measurements were administered just before the intervention, the post-tests were completed at the end of the intervention—that is, 6 weeks later—and finally, the follow-up tests were completed 8 weeks after the post-test.

### 2.3. Intervention

The interventional program emphasized promoting the developmental integration of emotions, feelings, as well as cognitive and behavioral skills. Due to the spread of students across multiple schools in the intervention group, cohorts of 3–5 students were created based on school proximity and were provided with training.

The lessons in the program were about: feelings, thinking (positive, negative), outcomes of negative thinking, coping strategies, strengths and weakness, problem solving, communication skills, social skills, negotiation, despising and ridiculing, and intra- and interpersonal skills. A review of resilience programs in the literature helped us to develop and adapt the program. Input included the Penn Resiliency Program (PRP) [24], Resourceful Adolescent program (RAP-A) [25], Promoting Alternative Thinking Strategies (PATHS) [29] and the Aussie Optimism Resilience-focused Program (AORSP) [26]. The objectives of lessons in this program were mostly influenced by AORSP, since it is based on Seligman’s theory of Positive Psychology. (This program is described in the Supplementary Material).

The lessons were delivered by experienced itinerant teachers. They had several hours of training by the researcher. Students in groups of 3, 4, and 5 (intervention group) received the instructions in 12 sessions of 75 minutes over 6 weeks. In each session, the lessons followed a common format: an introduction from the teacher (in which the lesson topic and objectives were stated), group activities (using picture books and telling stories, questions and answers), individual activities such as drawing, and finally a brief plenary/closure (in which learned points were reviewed).

### 2.4. Data Analysis

The Klomogorov-Smirnove Test was used to examine the normality of EI scores’ distribution. The Non-parametric Friedman Test was used to compare the changes taking place between three stages of measurements. The Cohen’s d effect size was calculated and classified as small (0.2–0.49), medium (0.5–0.79), or large (0.8 or more) [30].

## 3. Results

The participants consisted of 122 students with hearing loss; the M/F ratio was 3/2 (74 boys and 48 girls). The age range was 12–15, and the mean and standard deviation for the age of participants was 13.65 ± 1.00. There were no significant differences between mean age and sex ratio of intervention and control groups. Of all participants, 78 (51.6%) used hearing aids in one ear, 44 (36.1%) in both ears, and 8 (6.6%) students used cochlear implants. With using hearing aid, the corrected hearing thresholds in the better ear of 83 (68%) participants were within the mild, 24 (19.7%) in the moderate, and 15 (12.3%) in the severe range of hearing loss. The cause of hearing loss for 106 (86.89%) students was congenital and for 16 (13.11%) was acquired. Two students had a parent with hearing loss (1.6%).

The resilience intervention increased the resilience scores by 20 points in a score range of 0–100.

Table 1 and Table 2 show the emotional intelligence scores in both intervention and control groups, before and after the intervention, respectively. 

Table 1 shows that there is no significant difference in the Emotional Intelligence scores between intervention and control groups before the intervention. 

As Table 2 indicates, there is a significant difference between the control and intervention groups in emotional intelligence and its domains after intervention. The effect sizes of intervention were medium for Emotion Utilization (ES = 0.7) and large for Emotion Regulation (ES = 1.2), Emotional Appraisal and Expression (ES = 1.3), and Emotional Intelligence total score (ES = 1.5).

The follow-up emotional intelligence scores in the intervention and control groups are demonstrated in Table 3. 

As Table 3 demonstrates, there is a significant difference between the control and intervention groups in emotional intelligence and its domain remains 8 weeks after the end of intervention (follow up). The effect sizes for the intervention was large for emotional intelligence (ES = 1.6) and its domains: Emotion Regulation (ES = 0.8), Emotion Utilization (ES = 0.8), and Emotional Appraisal and Expression (ES = 1.7).

## 4. Discussion

This study investigated the effectiveness of a resiliency intervention program on the emotional intelligence of adolescent students with hearing loss. The results showed that the resilience training program enhanced the emotional intelligence of the students. There are reports of the success of such programs in promoting emotional intelligence in adolescents with and without hearing loss. The effectiveness of the program (PATHS) was examined for students with hearing loss. The results showed significant progress in emotional recognition skills, social problem-solving skills, and teacher- and parent-rated social competence [29].

The findings of this study indicated that the effect sizes were large for EI, Emotion Regulation, and Emotion Appraisal and Expression and medium for Emotion Utilization after intervention. There are studies on emotion focused interventions with different results. Claro et al. studied the effectiveness of ‘Cognitive Emotion Regulation’, a school intervention for high school students, aiming at emotional regulation skills [31]. The program was effective in improving cognitive emotion regulation and the effect size was ES = 0.35, which is lower than those we found for effects of our resilience-focused program on emotion regulation in the present study (ES = 1.2). In a meta-analysis of 213 Socio Emotional Learning interventions for school children, Durlak et al. revealed significant mean effects in improved social emotional skills (ES = 0.57), positive social behavior (ES = 0.24), and emotional distress (ES = 0.24) [32]. Another meta-analysis related to enhancing emotional intelligence (EI) indicated a moderate overall effect size for the impact of EI training, which was SMC pre–post = 0.51 and was the same for RCTs. The mean effect size of those interventions ranged from 0 to 2.13 [33], and in 4 studies they were higher than the observed effect size (ES = 1.5) in the present study. The positive effect of this intervention program on EI may be related to the characteristics of the activities and encouragements employed. This intervention was based on structured and interactive activities (playing games, drawings, reading stories), and exercises adapted to adolescents with hearing loss. In order to encourage participation, positive reinforcement such as giving complements and praise was used. Although we found no report studying the effects of resilience program on emotional intelligence, some research [15,16,23] has revealed that resilient individuals can effectively manage or regulate their emotions. Positive emotion increases the possibilities that individuals find positive meaning in stressful and negative situations, and individuals with high resilience would be hurt less by mental confusion and emotional problems [16,23]. They are emotionally calm, and cope with adverse conditions in an appropriate manner [34].

As the results indicate, for the intervention group, the scores for EI and its domains (Emotion Regulation, Emotion Appraisal and Expression, and Emotion Utilization) increased from pre intervention to post intervention. Scores for the Emotion Appraisal and Expression domain increased more than other domains of emotional intelligence. It is believed that children with hearing loss can be taught to identify and express emotions through training (using pictures, slides, role-playing, stories, art activities) [35,36], and in the present program, there were lots of such activities and practices that could lead to such an increase in this domain.

Our results show a slight drop in follow-ups of Emotion Utilization scores for both groups. This could be related to the timing of the follow-up test, which was close to the midterm school exams. Therefore, in that stressful time, they might experience some difficulties in the processing of emotional information. It is known that poor emotion information processing deforms emotion knowledge and degrades emotion utilization [37].

In this study, parents were informed about the aim and the activities done in each session by sending them an information sheet, and they were asked to follow them at home, but no assessments were scheduled for parental competence in following instructions. Further research could consider some structured practices and assessments in this regard, or the management of training sessions for parents along with adolescents.

Emotional intelligence is one of the top topics among mental health professionals for adolescents. Since one of the causes of mental health problems is the inability to manage emotional states, some research in this area is addressing the effects of EI interventional programs [38,39]. Schools are identified as an important place for supporting adolescent emotional health, and many classroom-based programs in schools are focused on emotional health interventions. The present program could be useful for adolescents and those with special needs, if some justifications based on their needs are made.

A limitation of the study was the use of self-reporting to measure emotional intelligence, and this could have affected the results. Future studies could be done using assessment instruments completed by parents/teachers and/or a professional observer. In this study, only students in mainstream schools were included, so the findings may not be generalizable to adolescents with hearing loss who study in special schools. Hopefully, future research will address this issue.

## 5. Conclusions

This study offers valuable information regarding the benefits of providing programs that target resiliency for emotional intelligence skills in adolescents with hearing loss. Children with hearing loss are at risk of emotional problems. Hopefully, the interventional programs in schools such as this program which includes interesting activities and practices adapted to the adolescents with hearing loss, would promote their emotional intelligence and resilience. These kinds of interventions might also stimulate the teacher’s sense for what excites students and captures their attention and curiosity for learning.

## Figures and Tables

**Table 1:** Emotional Intelligence comparison between intervention and control group (before intervention) just after the intervention.

EI Domain	Scoring Range	Control Group	Intervention Group	*p*-Value
		Mean ± SD	Mean ± SD	
Emotion regulation	0–50	32.92 ± 5.67	32.15 ± 4.32	NS *
Emotion Appraisal and Expression	0–65	38.38 ± 5.80	38.06 ± 4.40	NS
Emotion Utilization	0–50	31.08 ± 5.27	31.65 ± 4.04	NS
Total Emotional Intelligence score	0–165	105.64 ± 14.51	105.00 ± 9.37	NS

* NS = Not Significant.

**Table 2:** Emotional Intelligence comparison between intervention and control group (6 weeks after the day 1) just after the intervention.

EI Domain	Scoring Range	Control Group	Intervention Group	*p*-Value	Effect Size
		Mean ± SD	Mean ± SD		
Emotion regulation	0–50	32.30 ± 4.16	37.48 ± 5.18	<0.001	1.2
Emotion Appraisal and Expression	0–65	38.77 ± 3.94	44.16 ± 5.10	<0.001	1.3
Emotion Utilization	0–50	32.69 ± 3.50	35.30 ± 4.22	<0.001	0.7
Total Emotional Intelligence score	0–165	106.90 ± 9.27	120.79 ± 12.52	<0.001	1.5

**Table 3:** Emotional Intelligence comparison between intervention and control group, in follow up Measurement (8 weeks after the end of intervention).

EI Domain	Scoring Range	Control Group	Intervention Group	*p*-Value	Effect Size
		Mean ± SD	Mean ± SD		
Emotion regulation	0–50	33.51 ± 4.55	37.10 ± 3.44	<0.001	0.8
Emotion Appraisal and Expression	0–65	38.56 ± 3.16	44.02 ± 3.75	<0.001	1.7
Emotion Utilization	0–50	30.87 ± 3.58	33.75 ± 4.13	<0.001	0.8
Total Emotional Intelligence score	0–165	105.15 ± 8.26	118.56 ± 8.33	<0.001	1.6

## Supplementary Materials

The following are available online at https://www.mdpi.com/2227-9067/6/3/48/s1, Aussie Optimism Resilience-focused Program (AORSP) description.
