# Supplementary material for: The Effectiveness of a Resilience Intervention Program on Emotional Intelligence of Adolescent Students with Hearing Loss
Source: Children (Basel). 2019 Mar 21;6(3):48. doi: 10.3390/children6030048 (PMC6463041; doi:10.3390/children6030048)
Supplement: Supplementary file 1 [file children-06-00048-s001.pdf]

Supplementary

The intervention package consisted of two booklets; one for the itinerant teacher and the other one for the student. The itinerant's booklet consisted of 12 modules, each of which were begun with describing the aim and introduction of the module, the instruments (student's booklet, black or white board, for some sessions story books and etc.), time needed for each activity, the manual for teaching and doing activities with students, and a supplemental pamphlet for informing parents about what their child learned in that session and practices that parents could apply at home. The student's booklet was also consisted of 12 modules which included individual and group activities (such as answering the questions in the booklet, drawing, discussing the subject, playing games, role playing and etc.).

The program included 12 sessions:

Session 1; Feelings: The aim was helping students learn about the feelings (happy, sad, excited, angry, tired, scared, relax, jealous, safe, have a good feeling, board, confused). In the student's booklet, every feeling was shown by a picture with a short story and some questions (teacher's booklet had the answers and she/he could give an example to guide the students), such as what things can cause this feeling? (For example for happy feeling, party or being with a friend makes me happy), if somebody or you have this feeling what do you do? (For example smiling or laughing). For negative feelings, there was additional question; what could you do to change this feeling and feel good (for example the student can answer talk to somebody, watch TV, reading a book, calling friends). After answering the questions, students draw a face to show that feeling. At the end of session, each student role played a feeling and other students guess it, and if it was negative feeling they suggest how they can change that feeling.

Session 2; Feeling and thinking: The aim was that students learn about how feelings can impact the thinking, how our change of thinking can change our feelings, and how positive self-talk can help us. In this module, there were two tables; one consisted of different feeling and one various kinds of thinking. Two stories focusing on feelings and thinking were read and some questions were asked, such as what is her feeling? What does she think? If she changes her thinking, what is her feeling now? Do you think your thoughts can impact your feeling; give us an example. In the next activity, students looked at the thinking table and chose one (for example I cannot do my homework because it is hard to do), then saw feeling table and searched for those that can match it (sad, tired, angry, etc). Students were asked, how they could change that thinking and what happens (students could answer; I can do my homework with the help of others and feel happy). There were more practices such as short stories about negative thinking and feeling, also questions and discussion about how could they change those to positive thinking and feelings (some examples from students too).

Session 3; Abilities (strengths and weakness): The aim was to help students know that every individual has some strengths and weaknesses and no one can be completely good or completely

bad. A storybook about a crow which couldn't see his strengths and wanted to be someone else was read. Students were asked some questions. A sign of + (positive) and a sign of -(negative) was written on the board, and asked if they knew what were these signs and what did they think about them. They were asked to give some strengths of the crow and the teacher wrote them under + signs, and the same thing was done for the weaknesses and - sign. There was a discussion about them. Then, every student wrote an example of a good or positive behavior as strength and a negative behavior as a weakness. The teacher wrote them on the board. Also, the teacher drew two large circles; one with +sign on the center and one with negative sign. Several pieces of papers; each with one of the signs were in a box and every student chose one. When the student opened the paper, based on the sign, he/she read a couple of those strengths or weaknesses from the board and the teacher wrote them in the related circle. Again the teacher asked questions such as could a person has just good or positive characteristics and behaviors (or has just negative characteristics)? If you don't or can't do good things, do you think you are a bad person or just have some weaknesses? They had discussion.

Session 4; Coping: The aim was that students learn about the signs of stress, the approaches they could choose for coping and self-management. There are different reactions to changes that happen during the life. The teacher explained that some of the challenges are hard and students should know they can get help from the adults. Students were asked if they know what is stress and how could they cope? Students gave some examples. Again students were asked what things cause stress for them. Every student gave some answers and the teacher wrote them on the board. For every answer, teacher asked about student's feeling and also asked if they could control themselves in that situation, and how can they control it. The teacher also talked about the situations that can't be under control such as death of a close relative or friends, moving to another city and etc. Students were asked to give some more examples and what could they do about them. They discussed different strategies. Another subject was about alert signs. The teacher explained that with stress and anxiety our body shows signs like having high heartbeat. Students were asked to name some more of those signs and the teacher wrote them on the board. Every student was asked about the situations that one of those signs could happen for them and discuss how they could control it. The teacher talked about some other coping approaches such as thinking about good things, imagining the things they like or makes them happy, leaving the situation and does something likable, amusement, and relaxing (practicing body relaxation). The teacher gave an example for each and students gave some more examples and discus different ways of coping and adjustments.

Session5; Problem solving: The aim was that students recognize a problem, find effective ways to solve it, evaluate those and choose the best one. The teacher explained that everyone has different problems in life. Students should learn they could solve their problems or get help from adults. The teacher explained about some times they could use self-talk (for example if someone didn't get a good grade, he/she could tell her/his self don't worry I could think of something or get help (positive self-talk), or he/she could say he/she couldn't do anything(negative self-talk).

The teacher and students worked on some more examples of different situations and discuss what could be the negative self-talks and what could be the positive ones. Also the teacher talked about stages of problem solving: (1) recognizing the problem in which the teacher gave a few examples and students determined the problem for each situation. (2) Thinking about different ways of solving a problem in which students discussed the ways for each problem. (3) Choosing the best way so that the result would have a positive impact. The teacher explained about solving approaches: Sad, mad, and glad ways of solving a problem. In a situation such as somebody laugh at you, you hit him/her (mad way) or leave the situation (glad way), what are the impacts of each? The teacher presented more examples of different situations and students discuss various ways and their impacts.

Session 6; Communication skills: The aim was that students learn about the importance of verbal and oral (facial and body) languages, and listening skills. Every two students chose a subject and talked about it. When one of them talked, the other one just listened and at the end he/she could ask questions. They changed their places and continued the practice. The teacher explained the importance of body language and how could the listener notice the body language of the talker. The listener should acknowledge some rules: stand in front of the talker; look at his/her eyes, face, and voice. Students practiced with different scenarios. In the next activity, the teacher wrote different situations on the board and every two students practiced communication orally or verbally considering the given rules. They discuss how they could use those skills in different situations such as in school or classroom in interaction with their friends or teachers.

Session 7; Negotiation: The aim was that students learn the appreciation of other peoples' perspectives, know about the situations that they could use negotiation, develop their social, communicational, and problem solving skills. The teacher read a story, "We can solve our problems with having conversation". Students answered the questions and discussed how the characters in the story spoke and negotiated for solving their problem. Students suggested some examples of negotiation situations. Every two students chose a situation and role played the conversation and negotiation skills. After finishing this activity, the teacher explained about the stages of negotiation process: (1) you: tell your counterparts what you want (consider these points; did you tell him directly or indirectly? Was it fair), (2) they: listen to her/him what does he/she wants (did you listen very well? do you know what he/she wants), (3) Negotiate: remember that you have different choices (for example; I can do this if you…). Various situations were written on the board and students practiced this skill based on the negotiation stages. These practices helped students to develop social, communication, and problem solving skills too.

Session 8; social skills (making good relationships): The aim was that students learn about how they could express their feelings, how could they get help when needed, straight talk, and how to make and reinforce a good relationship. Different ways of communication or making the relationships could impact the mental health of students. The teacher explained about two important things that the listener should do: "listen with interest" and "show the talker that you

are interested in him/her". Students were asked if they saw two people talking about one subject (such as football), how they talked and reacted. Students suggested some more examples of such situations and discussed their ways of talking and voice tones, their facial changes, and reactions. In the next activity, the teacher sat face to face with a students and the student talked about a subject and teacher listened to him/her considering some rules (looking at student, smiling, shaking head for being agreed, not interrupted his/her talk and waited for him/her to finish to ask questions or continued the conversation). Then asked students what did I do to show the other person that I liked him/her. For more practice every two students sat in front of each other, chose a subject and practiced based on those rules. Another item was "interesting characters". Children and adults usually are attracted to special and interesting characters of others. Students were wrote some interesting characteristics of themselves (for example I write very well or I draw very good and etc.), and discuss them. "Friendly habits" was another subject. Some questions such as were you in a situation that your friend asked you to do something or did you go somewhere and you didn't want to go? The teacher had some more actual examples and explained how students could use straight talk and say "No" politely. They should consider some rules such as saying "No" with smiling, peace and quiet tone of voice, and being relaxed and not get angry. He/she explained that they could tell the other person such things; I don't feel good about this, let me test it or ask somebody for guidance, please don't ask me again or maybe next time. Another explained subject was that sometimes for making a relationship, a person may say good things about you, and you should appreciate it. Students practiced different situations by role playing.

Session 9; Social skills: The aim was that students know about the benefits of friendly habits, recognize the relationship between thinking and feelings use of social skills to express feelings, appreciate others' feelings, and getting help when needed. The teacher asked students if they were in a situation in which they wanted to say "no" but under pressure they said yes. Students suggested some examples and talked about them. The teacher explained about the three stages of decisive relationship with direct talk. (1) Describe the situation (when you want something from me), (2) Express your feeling (I feel bad or bothered), (3) Tell what you want (please don't ask me again). For practice, there were examples of different situations in students' booklet which the teacher wrote them on the board. Every two students chose one, and role play it. After finishing each role play, other students gave feedback and discussed it more. Also, students gave some more examples and they talked about it. Another subject was decision making in which the teacher had an example and some questions. Then, students suggested examples of situations they had to make decision. They discussed them and the teacher made some important points to consider: don't hurry in making a decision, think about the results of the decision and the impact of it, if the decision making is hard and an individual doesn't know what to do, he/she should get help from trusted friends or adults. Students practiced by discussing various situations in which a person has to make a good decision.

Session 10; belittlement and ridiculing: The aim was that students know about belittlement and ridiculing, understanding that others' talking about them isn't necessary true, not belittle or ridicule other people, and what can a person do in case of belittling and ridiculing. The teacher had examples of belittlement (such as a father tells his child that he can't do anything right), and ridiculing (a student answers a question in class and some of students laugh at him/her). Students were asked about the meaning of each concept and explained about them. A short story about the belittling was read for students and some questions were asked for discussion. The teacher made some points: when you are in such situations don't react immediately and get angry because they want to make you angry and do something bad, have positive self-talks; are they right about me, I don't like their talking but I don't get angry or sad because I know they are not right, if I get angry I did what they wanted me to do. Students presented some examples and role played them. The same things were done for ridiculing. Additional activity was done in this part in which students draw an ugly face and put it on their face as a mask. The teacher asked if other students could say different words about the faces (students answered; horrible, retarded, ugly, etc.), how did they feel if somebody used these words for them. Students practiced positive self-talks and how could they handle such situations by discussion and role playing.

Session 11;   Control the negative thoughts: The aim was to demonstrate how to come to terms with negative thoughts and feelings and control them. Sometimes students have negative thoughts or others cause them to think or feel negatively. The teacher asked students if they had an experience in which they couldn't answer their teacher's question in class; what their thought were. What were their feelings? The teacher wrote their answers and more negative thoughts and feelings on the board. She/he explained if they could change those negative thoughts, they could have different feelings. There were some more examples of negative thoughts in various situations in which students had discussion about them and learned how they could change those negative thoughts and feeling to positive ones. Another subject was the interactions and relationships and how can students adjust themselves when others have an inappropriate behaviors with them. The teacher gave an example of about him/herself and what didn't she/he like in that situation (when I was talking, somebody laughed at me). Students suggested some more examples of what didn't they like, and the teacher wrote them on the board and talked about them. Students were asked, if someone ridiculed them or used abusive language, what could they do? The answers were written on the board and the teacher explained about more things they could do. Students have role played the situations.

Session 12; Positive and negative interpersonal skills: The aim was that students learn to discriminate between positive and negative interpersonal skills. The teacher presented an example of a situation: 'you and your friend went for shopping to buy a gift; did you talk to each other about the gift you wanted to buy? Were both of your ideas the same? If you've had different idea, did you accept your friend's idea and forgot your own. After students answered the questions and talked about them, the teacher read a story; "why are you fighting". Students answered some questions about the story and discussed what they learned from the story. The

teacher explained about positive and negative interactions and some interactions could be both negative and positive. She/he gave examples of different situations with some questions. Students had more discussion.
